# Population size in QTL detection using quantile regression in genome-wide association studies

**DOI:** 10.1038/s41598-023-36730-z

**Published:** 2023-06-13

**Authors:** Gabriela França Oliveira, Ana Carolina Campana Nascimento, Camila Ferreira Azevedo, Maurício de Oliveira Celeri, Laís Mayara Azevedo Barroso, Isabela de Castro Sant’Anna, José Marcelo Soriano Viana, Marcos Deon Vilela de Resende, Moysés Nascimento

**Affiliations:** 1 Federal Institute of Education, Science and Technology of Mato Grosso, Sorriso, Mato Grosso Brazil; 2 Rubber Tree and Agroforestry Systems Research Center, Campinas Agronomy Institute (IAC), Votuporanga, São Paulo, Brazil; 3 Department of General Biology, Federal University of Viçosa, Viçosa, Minas Gerais Brazil; 4 Brazilian Agricultural Research Corporation, Embrapa Coffee, Brasília, DF Brazil; 5 Present Address: Department of Statistics, Federal University of Viçosa, Av. Peter Henry Rolfs, S/N, Campus Universitário, 36570.900, Viçosa, Minas Gerais Brazil

**Keywords:** Genetics, Agricultural genetics, Genetic association study, Genetic markers, Plant breeding

## Abstract

The aim of this study was to evaluate the performance of Quantile Regression (QR) in Genome-Wide Association Studies (GWAS) regarding the ability to detect QTLs *(Quantitative Trait Locus)* associated with phenotypic traits of interest, considering different population sizes. For this, simulated data was used, with traits of different levels of heritability (0.30 and 0.50), and controlled by 3 and 100 QTLs. Populations of 1,000 to 200 individuals were defined, with a random reduction of 100 individuals for each population. The power of detection of QTLs and the false positive rate were obtained by means of QR considering three different quantiles (0.10, 0.50 and 0.90) and also by means of the General Linear Model (GLM). In general, it was observed that the QR models showed greater power of detection of QTLs in all scenarios evaluated and a relatively low false positive rate in scenarios with a greater number of individuals. The models with the highest detection power of true QTLs at the extreme quantils (0.10 and 0.90) were the ones with the highest detection power of true QTLs. In contrast, the analysis based on the GLM detected few (scenarios with larger population size) or no QTLs in the evaluated scenarios. In the scenarios with low heritability, QR obtained a high detection power. Thus, it was verified that the use of QR in GWAS is effective, allowing the detection of QTLs associated with traits of interest even in scenarios with few genotyped and phenotyped individuals.

## Introduction

The world's population reached 7.7 billion inhabitants in 2019 and may reach 9.7 billion by 2050^1^. To the increase in population is added the growing concern about environmental impacts and the limitations of arable areas, which culminates in the demand for increased productivity of agronomic species^2^. In recent years, it is estimated that about 50% of the increase in productivity of several species was driven by genetic breeding, which has been seeking new strategies to obtain more adapted, resistant, and productive cultivars^3,4^.

In this context, genome-wide association studies (GWAS) have been conducted in order to identify genetic variations that may be associated with phenotypic traits of interest^5–9^. The potentials of GWAS have already been successfully explored in traits of economic interest and in different crops, such as barley^10,11^, maize^12–14^, soybean^15,16^, rice^17–20^, wheat^21–23^ e arabica coffea^24–26^.

In GWAS, a classic and widely used statistical method is single markers regression. This method estimates the individual effect of each marker on the phenotype of interest, and, subsequently, multiple hypothesis tests are performed in order to detect which marker effects are statistically significant^27^. When the correction for population structure is added to the single markers regression model, this model is called General Linear Model (GLM)^28^.However, the estimation of parameters via single markers and GLM are based on conditional means, which may be inadequate when the errors do not follow a normal distribution^29^ and in the presence of heteroscedasticity. An alternative and still little explored methodology for GWAS studies is Quantile Regression (QR)^30^. This methodology, unlike methods based on means, allows adjusting regression models for different levels (quantiles) of the distribution of the phenotype of interest, does not require assumptions about the error distribution, and is robust to discrepant points^31^. QR has already been successfully applied in GWAS studies on real data by^32^ for traits related to the flowering time of common beans. These authors evaluated 80 common bean genotypes and 384 SNP markers (*Single Nucleotide Polymorphism*) in order to identify genomic regions for three phenological traits. As a result, the authors found no significant associations using the General Linear Model. In contrast, when using QR at the extreme quantile (τ = 0.10), it was possible to detect 7 significant associations between SNPs and the phenological traits studied. In this study, the number of available genotypes was relatively small for GWAS studies, but it was still possible to detect significant associations using QR in this setting.

Although QR has already been applied to real data sets and has obtained interesting and promising results, the effect of population size on the ability to detect QTLs *(Quantitative Trait Locus)* has not yet been evaluated. To this end, it is possible to use data simulation since this strategy aims to reproduce the conditions of a biological system, facilitating the understanding of its real functioning and allowing prediction of the performance and recommendations before starting field studies^33,34^. In addition, simulation studies are especially convenient for testing and comparing methodologies because they demand fewer resources, time, human efforts, and the possibility of replication, thus generating greater efficiency in inferences^34,35^.

In view of the above, this study evaluated the use of QR in GWAS regarding the power of QTL detection through SNP markers for simulated data with different levels of heritabilities, trait loci, and population sizes. The results of QR were compared with those obtained by GLM.

## Material and methods

Aiming to access the power of QTL detection and false positives rates in a genome-wide association study was performed a simulation study.

### Genome and simulated populations

An advanced generation composite was obtained from two random mating populations in linkage equilibrium, which were crossed to generate a population of 5,000 elements from 100 families using linkage disequilibrium (LD), subjected to five generations of random mating without mutation, selection, or migration.

From the advanced generation of the composite, 1000 individuals from the same generation and from 20 families of full siblings, each consisting of 50 individuals, were simulated. The simulated genome was composed of ten chromosomes with a size of 200 centimorgans (cM) each and comprised 2000 bi-allelic single nucleotide polymorphisms (SNPs) separated by 0.1 cM across the ten chromosomes. The LD value in a composite population is ${\Delta }_{ab} = \left( {\frac{{1 - 2\theta_{ab} }}{4}} \right)\left( {p_{a}^{1} - p_{a}^{2} } \right)\left( {p_{b}^{1} - p_{b}^{2} } \right)$, where a and b are two SNPs, two QTLs, or one SNP and one QTL, θ is the frequency of recombinant gametes, and $p^{1}$ and $p^{2}$ are the allele frequencies in the parental populations (1 and 2). The LD value depends on the allele frequencies in the parental populations. Thus, regardless of the distance between the SNPs and/or QTLs, if the allele frequencies are equal in the parental population, Δ = 0. The LD is maximized $\left( {\left| {\Delta } \right| = \,0.25} \right)$ when θ = 0 and $\left| {p^{1} - p^{2} } \right| = 1$. In this case, the LD value is positive with coupling and negative with repulsion^36^.

### Simulation of traits and the phenotypic values

Two genetic architectures were simulated, representing different scenarios, with heritabilities of 0.30 and 0.50 and with 100 and 3 numbers of quantitative trait loci (QTLs), distributed randomly in the regions covered by the SNPs. The first scenario follows the infinitesimal model and the other (second scenario) with three major effects genes accounting for 50% of the genetic variability. For the former, to each of 100 QTLs one additive effect of small magnitude on the phenotype was assigned (under the Normal Distribution setting). For the latter, small additive effects were assigned to the remaining 97 loci. The effects were normally distributed with zero mean and variance, allowing the desired heritability level. The phenotypic value was obtained by adding to the genotypic value a random deviate from a normal distribution $N\left( {0,\sigma_{e}^{2} } \right)$, where the variance $\sigma_{e}^{2}$ was defined according to two levels of broad-sense heritability, 0.30 and 0.50.

The data set was simulated using the Real Breeding program^37^. More information can be found detailed in^38^.

Subsequently, in order to evaluate the effect of population size reduction, populations were defined with numbers of individuals ranging from 1,000 to 200 individuals. According to^39^, 200 individuals are considered as being sufficient for the construction of reasonably accurate genetic maps. A random reduction of 100 individuals was defined in each scenario, respecting the proportionality of individuals removed from each family. Thus, in all, thirty-six distinct scenarios were evaluated. These scenarios correspond to the combination of two levels of heritability, two genetic architectures, and nine variations in population size.

### Linkage disequilibrium

A linkage disequilibrium (LD) analysis was performed to determine the markers associated with QTLs. Specifically, the LD decay pattern between marker pairs across the genome was obtained using a figure in which the square values of the correlation coefficient r^2^ were plotted against the genetic distance between markers (in cM). Subsequently, a local polynomial regression (LOESS)^40–42^ was fitted to the data and a horizontal straight line was plotted with a critical value of *r*^2^ = 0.20^43,44^. The window distance, defined as the intersection of the fitted LOESS curve and the horizontal straight line, will be used to determine which markers are associated with QTLs. Thus, all markers that distance the value of the window obtained (depending on the scenario evaluated) in relation to each QTL are considered as markers associated with the QTLs. The square of the correlation coefficient $\left( {r^{2} } \right)$ was estimated using the *LD.decay* function of the *sommer* package^45^ and the fit of the polynomial regression model using the *loess* function, both from the R software^46^.

### Genome-wide association study

To perform the genome-wide association analysis, first, the correction for population structure was performed through principal component analysis (PCA) of the genomic relatedness matrix (G)^20,47,48^. The number of principal components adopted was obtained using STRUCTURE 2.3.4 software^49^, selecting 300 markers in linkage equilibrium, aiming to ensure that these markers are not associated. A cluster number (K) ranging from 1 to 21 was tested, with ten independent replicates for each K value. In order to identify the optimal number of K, 10,000 iterations were run, with 1,000 burn-in. Then, the *∆K* index^50^ implemented in Structure Harvester software^51^ was calculated to determine the choice of the most likely value of K. Subsequently, the K first principal components (CP) were used as fixed effect covariates in the GWAS model.

The GWAS model was defined by:

$$Y = \mu + \alpha_{j} SNP_{j} + \mathop \sum \limits_{k = 1}^{K} \beta_{k} CP_{k} + \varepsilon$$

where *Y* is the vector of phenotypic information; μ is the population mean; $\alpha_{j}$ is the effect of the j-th marker considered as fixed, $j = 1, \ldots , 2000$; $SNP_{j}$ is the incidence vector of the j-th SNP marker; $\beta_{k}$ is the fixed effect of the k-th principal component, adjusted as a covariate; $CP_{k}$ is the vector of the k-th principal component; $\varepsilon$ is the vector of random errors. The vector $\theta = \left[ {\mu ,\alpha_{j} ,\beta_{1},...,\beta_{k} } \right]^{^{\prime}}$ represents the unknown parameters, being estimated by means of QR and the GLM.

The methods estimate the individual effect of each marker on the phenotype of interest and then perform multiple hypothesis tests in order to detect which marker effects are statistically significant. The parameters were estimated via QR for different levels (quantiles) of the distribution of the phenotype of interest^30,32^. This methodology consists of estimating the parameters at the $\tau$ quantile by solving the following optimization problem:

$$\hat{\theta }_{\tau } = \arg \min_{{\hat{\theta }_{\tau } }} \left[ {\mathop \sum \limits_{i = 1}^{N} \rho_{\tau } \left| { \varepsilon_{i} } \right|} \right],$$

where $\tau \in \left( {0,1} \right)$ indicating the quantile of interest, N indicates the population size evaluated, and ρ_τ_ (·), denoted *check* function by^30^, is defined by:

$$\rho_{\tau } \left( { \varepsilon_{i} } \right) = \left\{ {\begin{array}{*{20}l} {\tau \varepsilon_{i} ,} \hfill & {if\ \varepsilon_{i} \ge 0,} \hfill \\ {\left( {\tau - 1} \right) \varepsilon_{i} ,} \hfill & {if\ \varepsilon_{i} < 0} \hfill \\ \end{array} } \right..$$

In this study, three quantiles (τ = 0.10, 0.50 and 0.90) were evaluated. For model fitting, the *rq* function from the *quantreg* package^52^ of the R software was used. The individual coefficients (effects) of each marker are estimated by summing the weighted absolute errors. For estimation, it is necessary to use linear programming algorithms. One of the methods used is the Simplex Method^53^.

The parameters were also estimated using GLM. This methodology consists of estimating the parameters in average terms and solving the following optimization problem:

$$\hat{\theta } = \arg \min_{{\hat{\theta }}} \left[ {\mathop \sum \limits_{i = 1}^{N} \varepsilon_{i}^{2} } \right].$$

For model fitting, the individual coefficients (effects) of each marker were estimated by minimizing the sum of squared errors by the ordinary least squares method using the GAPIT R package^54^ of the R software^46^.

### Hypothesis testing

After estimating the effects of individual markers through QR and GLM, multiple *t-student* tests were performed according to the methodology used, in order to analyze the existence of significant associations between the marker and the phenotype of interest. In the general linear model, the standard error estimate used was the usual, while in the quantile regression it was based on *rank*^53,55,56^. However, due to the high density of markers, performing multiple tests can lead to an increase in false positive associations^27^. An alternative to controlling this rate is the *False Discovery Rate* (FDR)^57,58^. One way to consider the FDR in hypothesis testing is through a correction in the p-value associated with the test, called the q-value^59^. In this study, a significance level of 0.01 ($\alpha =1{\% }$) corrected by the FDR was used.

### Comparison between methodologies

In order to evaluate the efficiency of the analyzed methodologies, the QTL detection power and the false positive rate were calculated and defined below: i) The power of QTL detection corresponds to the proportion of pre-established windows (intervals) (by means of LD analysis) that contain at least one marker considered significant by means of the statistical methods evaluated. ii) The false positive rate corresponds to the ratio between the number of markers that were significant by the evaluated statistical methods and are not associated with QTLs and the number of markers that are not associated with QTLs.

## Results and discussion

### Population structure

**Figure 1:**
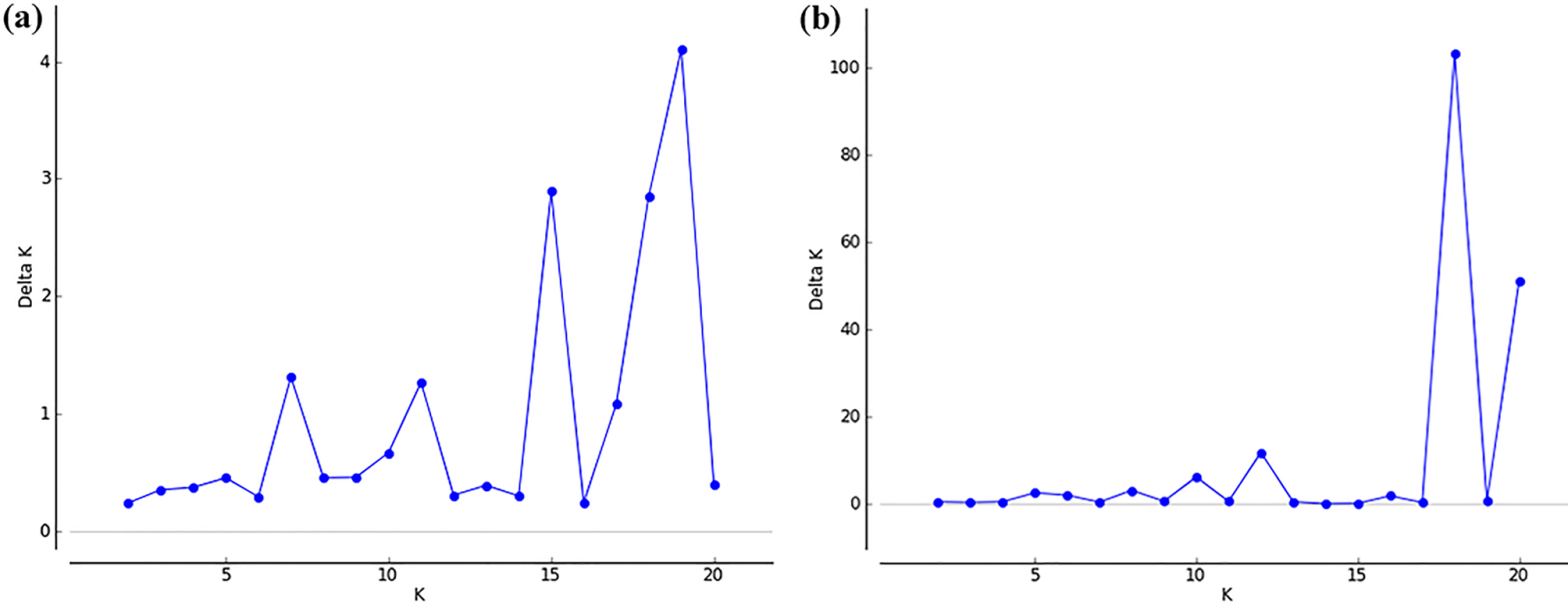
Graph ∆K versus number of clusters K. (**a**) Scenario with 3 QTLs. (**b**) Scenario with 100 QTLs.

According to the method of^50^, ∆K was plotted against the number of clusters (k). The maximum value of ∆K occurred at K = 19 and K = 18 for the scenarios of 3 QTLs and 100 QTLs, respectively (Fig. 1). Thus, 19 and 18 principal components were used as covariates in the GWAS analyses. According to the principal component analysis, 19 and 18 PCs accounted for explanation percentages of the variance present in the genotypic data between 85 and 96%, depending on the scenario evaluated. This result is in agreement with the simulated data of this study, where populations were simulated from 20 full sib families.

### Linkage disequilibrium

**Figure 2:**
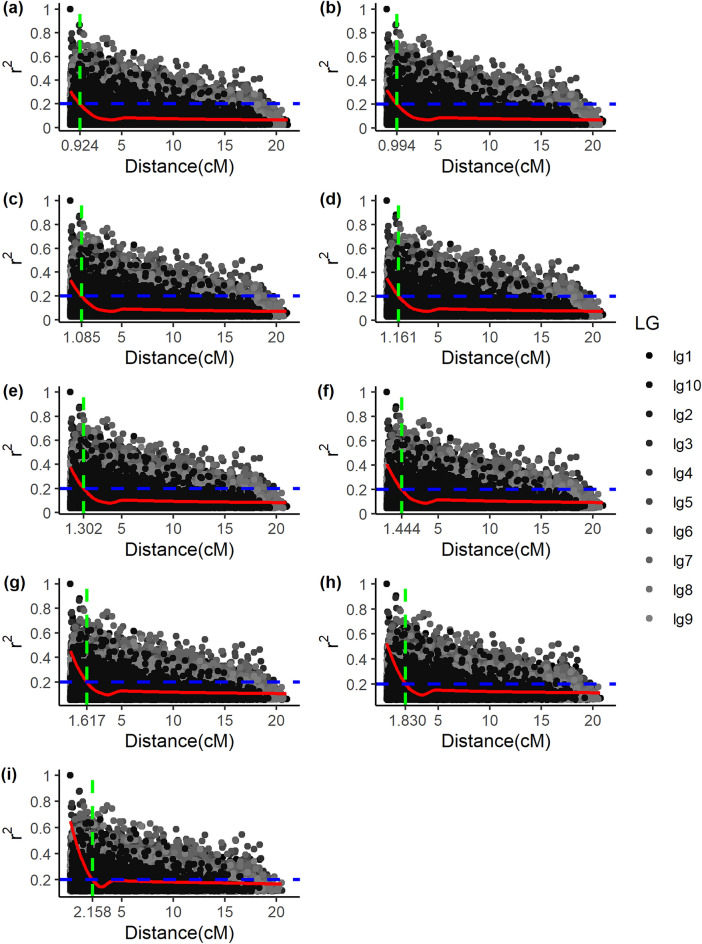
Decay of linkage disequilibrium (r^2^) as a function of genetic distance in the 10 linkage groups in the scenario with 3 QTLs. (**a**) Scenario: 1000 individuals (**b**) Scenario: 900 individuals (**c**) Scenario: 800 individuals (**d**) Scenario: 700 individuals (**e**) Scenario: 600 individuals (**f**) Scenario: 500 individuals (g) Scenario: 400 individuals (**h**) Scenario: 300 individuals (**i**) Scenario: 200 individuals.

**Figure 3:**
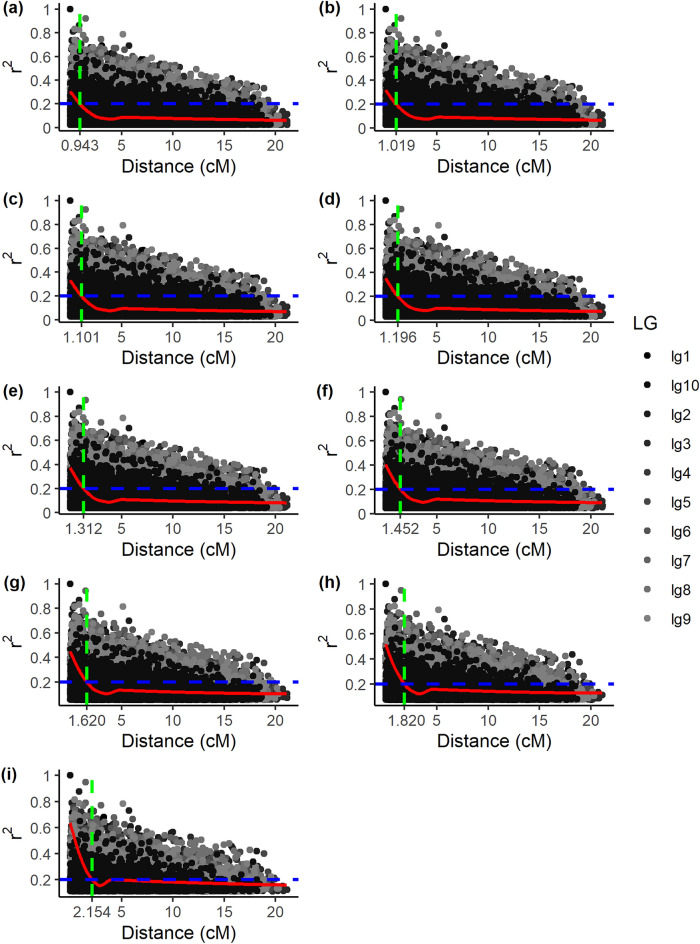
Decay of linkage disequilibrium (r^2^) as a function of genetic distance in the 10 linkage groups in the scenario with 100 QTLs. (**a**) Scenario: 1000 individuals (**b**) Scenario: 900 individuals (**c**) Scenario: 800 individuals (d) Scenario: 700 individuals (**e**) Scenario: 600 individuals (**f**) Scenario: 500 individuals (**g**) Scenario: 400 individuals (**h**) Scenario: 300 individuals (**i**) Scenario: 200 individuals.

The LD was calculated for all marker pairs in the same linkage group by means of r^2^. Figures 2 and 3 graphically represent the decay of LD as a function of genetic distance according to the number of QTLs evaluated. The critical value of $r^{2} = 0.20$ was adopted, which according to^43^, it is expected that values of $r^{2} < 0.20$, the LD is corrupted, that is, there is a tendency of linkage equilibrium between the markers. The intersection of the LOESS curve with the horizontal straight line $\left( {r^{2} = 0.20} \right)$ for the scenarios (different population sizes) of 3 QTLs, with a reduction in the number of individuals from 1000 to 200, was 0.924 cM, 0.994 cM, 1.085 cM, 1.161 cM, 1.302 cM, 1.444 cM, 1.617 cM, 1.830 cM and 2.158 cM, respectively (Fig. 2).

As for the scenario with 100 QTLs, the intersections obtained were: 0.943 cM, 1.019 cM, 1.101 cM, 1.196 cM, 1.312 cM, 1.452 cM, 1.620 cM, 1.820 cM, and 2.150 cM (Fig. 3).

After obtaining these values, it was determined that all markers that are less than the distances mentioned above (depending on the scenario evaluated) from each QTL are considered as markers associated with the QTLs.

### Genome-wide association

The general linear model obtained a low power of detection of QTLs in all scenarios evaluated (Table 1). In the scenarios with 3 QTLs, regardless of heritability and population size, this methodology showed power values equal to or less than 0.03 (Table 1). In the scenarios with 100 QTLs with 1000 individuals and a heritability of 0.30, the GLM obtained a power of detection on average of 0.21 ± 0.07 and with heritability 0.50, the power of detection was on average 0.56 ± 0.09. As the population size was reduced, the detection power was reduced until it reached zero in all scenarios evaluated (Table 1). This result was already expected and can be corroborated by several studies in the literature. For example, in the study by^60^, in which the authors evaluated the effect of population size in GWAS, considering data from barley germplasm. In this study, the authors used a base population consisting of 766 individuals, and population size reduction was achieved by random resampling without replacement, forming populations with 96, 192, 288, 384, 480, 576, and 672 individuals, and observed that the detection power of QTLs decreased according to population size reduction^61^. Also evaluated the power of GWAS to identify true significant associations using simulated *Arabidopsis* data set with 200, 400, and 800 individuals. As a result, the authors observed that the power of identifying true associations decreased as the number of individuals decreased. In addition to these,^62^evaluated the influence of sample size in GWAS using simulated data from a Chinese soybean germplasm population consisting of 200, 400, 600, and 800 individuals randomly sampled from an ideal base population. As a result, the authors observed that the detection power of true significant associations decreased, and the false positive rate increased with decreasing sample size. Furthermore, according to^63^ and^64^, the efficiency of GWAS requires large population sizes.

**Table 1:** Means and standard errors (10 replicates) of QTL detection power against two methodologies.

N^o^. QTL	h^2^	Methods	Population size								
			1000	900	800	700	600	500	400	300	200
3	0.30	QR (0.10)	1.00 ± 0.00	0.97 ± 0.03	1.00 ± 0.0	1.00 ± 0.00	1.00 ± 0.00	1.00 ± 0.00	1.00 ± 0.00	1.00 ± 0.00	1.00 ± 0.00
		QR (0.50)	0.80 ± 0.07	0.90 ± 0.05	0.93 ± 0.04	0.96 ± 0.03	1.00 ± 0.00	1.00 ± 0.00	1.00 ± 0.00	1.00 ± 0.00	1.00 ± 0.00
		QR (0.90)	1.00 ± 0.00	1.00 ± 0.00	1.00 ± 0.00	1.00 ± 0.00	1.00 ± 0.00	1.00 ± 0.00	1.00 ± 0.00	1.00 ± 0.00	1.00 ± 0.00
		GLM	0.03 ± 0.03	0.03 ± 0.03	0.03 ± 0.03	0.03 ± 0.03	0.00 ± 0 .00	0.00 ± 0.00	0.00 ± 0.00	0 .00 ± 0.00	0.03 ± 0.03
	0.50	QR (0.10)	0.87 ± 0.07	0.90 ± 0.05	0.93 ± 0.04	0.93 ± 0.06	1.00 ± 0.00	1.00 ± 0.00	1.00 ± 0.00	1.00 ± 0.00	1.00 ± 0.00
		QR (0.50)	0.70 ± 0.10	0.70 ± 0.10	0.50 ± 0.06	0.77 ± 0.05	0.90 ± 0.05	1.00 ± 0.00	1.00 ± 0.00	1.00 ± 0.00	1.00 ± 0.00
		QR (0.90)	0.80 ± 0.09	0.97 ± 0.03	1.00 ± 0.00	1.00 ± 0.00	1.00 ± 0.00	1.00 ± 0.00	1.00 ± 0.00	1.00 ± 0.00	1.00 ± 0.00
		GLM	0.03 ± 0.07	0.23 ± 0.07	0.23 ± 0.07	0.23 ± 0.07	0.20 ± 0.07	0.07 ± 0.04	0.03 ± 0.03	0.03 ± 0.03	0.00 ± 0.00
100	0.30	QR (0.10)	0.92 ± 0.02	0.97 ± 0.02	0.97 ± 0.02	1.00 ± 0.00	1.00 ± 0.00	1.00 ± 0.00	1.00 ± 0.00	1.00 ± 0.00	1.00 ± 0.00
		QR (0.50)	0.54 ± 0.09	0.72 ± 0.07	0.82 ± 0.05	0.92 ± 0.03	0.96 ± 0.03	1.00 ± 0.00	1.00 ± 0.00	1.00 ± 0.00	1.00 ± 0.00
		QR (0.90)	0.95 ± 0.02	0.98 ± 0.01	1.00 ± 0.00	1.00 ± 0.00	1.00 ± 0.00	1.00 ± 0.00	1.00 ± 0.00	1.00 ± 0.00	1.00 ± 0.00
		GLM	0.21 ± 0.07	0.00 ± 0. 00	0.00 ± 0.00	0.00 ± 0.00	0.00 ± 0.10	0.00 ± 0.00	0.0 ± 0.00	0.0 ± 0.00	0.0 ± 0.00
	0.50	QR (0.10)	0.61 ± 0.06	0.77 ± 0.05	0.78 ± 0.07	0.93 ± 0.03	0.98 ± 0.01	1.00 ± 0.00	1.00 ± 0.00	1.00 ± 0.00	1.00 ± 0.00
		QR (0.50)	0.15 ± 0.06	0.23 ± 0.06	0.31 ± 0.08	0.57 ± 0.07	0.72 ± 0.06	0.85 ± 0.04	0.94 ± 0.02	1.00 ± 0.00	1.00 ± 0.00
		QR (0.90)	0.55 ± 0.06	0.64 ± 0.07	0.66 ± 0.07	0.85 ± 0.04	0.93 ± 0.02	0.98 ± 0.01	1.00 ± 0.00	1.00 ± 0.00	1.00 ± 0.00
		GLM	0.56 ± 0.09	0.07 ± 0.02	0.03 ± 0.01	0.04 ± 0.02	0.01 ± 0.01	0.01 ± 0.01	0.01 ± 0.01	0.01 ± 0.01	0.00 ± 0.00

*Nº QTL:* number of loci controlling the trait, *h*^2^ : heritability, *QR:* quantile regression, *GLM:* general linear model.

However, the pattern reported by the authors mentioned above and those observed here for the GLM was not observed when using the QR models. In general, the QR, in all scenarios evaluated, obtained high detection power (Table 1). Additionally, unlike the results obtained using GLM, the detection power of QTLs did not reduce with the decrease in population size (Table 1). This result may be related to the way in which the standard error is calculated by the two methodologies. In the GLM, the standard error estimate used was the usual one, while in the QR it was based on the rank statistic. The rank statistic is greatly influenced by the sample size^53,55^. Thus, the statistic of the test used generally presents higher values and, therefore, a greater number of QTLs being considered significant.

In scenarios with 3 QTLs, at quantiles of 0.10 and 0.90, regardless of heritability and population size variation, QR detected almost all simulated QTLs (Table 1). As for the scenarios with 100 QTLs, QR at the extreme quantiles (τ = 0.10 and 0.90) obtained higher or equal QTL detection power when compared to QR (τ = 0.50) (Table 1). In terms of population size, independent of heritability and quantile evaluated, QR detected all QTLs of interest considering population sizes equal to that of 200 and 300 individuals to QR (Table 1).

In general, the use of QR obtained a high QTL detection power independent of the population size, and especially in the extreme quantiles. This result is reasonable since QR uses the same idea of sampling for extremes^65^. Sampling extreme phenotypes samples individuals at the extremes in the hope that rare causal variants will be enriched among them^32^. However, unlike the extreme phenotype sampling approach, the use of QR does not require any assumptions about the distributions of traits, is robust to outliers, and uses all individuals in the estimation process, avoiding some problems related to extreme phenotype sampling, as an example, sampling bias and the assumption of normality^31,32^.

The detection of significant SNPs with a small population size and at the extreme quantile has already been observed by^32^. The authors evaluated 80 genotypes and 384 SNP markers of common bean, aiming to identify genomic regions for three phenological traits (Days to first flowering-DPF; Days to flowering-DTF; and Days to end of flowering-DFF). As a result, the authors found no significant associations using GLM. On the other hand, when using QR at the 0.10 quantile, one and six significant SNPs were found for DPF and DTF, respectively. Although the work of^66^ and^67^ was not conducted in the context of genome-wide association, the authors also evaluated the performance of QR on simulated data set with small population sizes and concluded that QR is a robust technique in these situations. This result is very promising in breeding programs that have a reduced number of available genotypes.

Regarding the rate of false positives, we have found that the GLM, in all scenarios evaluated, presented low values for this rate. This result may be related to the low detection power of QTls by this methodology (Table 2). The false positive rate obtained by the QR methodology is relatively low in the scenarios with a higher number of individuals. QR (τ = 0.50) was the methodology that presented lower false positive rates. In scenarios where the QR detection power in the three quantiles evaluated was equal, the QR (τ = 0.50) showed better results than in the extreme quantiles QR (τ = 0.10 and 0.90) since the false positive rate was lower (Table 2). Regarding the reduction in the number of individuals, the false positive rate increased substantially according to the reduction in population size, a result that may be related to the observed increase in the number of QTLs detected in these scenarios.

**Table 2:** Averages and standard errors (10 repetitions) of the false positive rate against two methodologies.

Nº. QTL	h^2^	Métodos	Population size								
			1000	900	800	700	600	500	400	300	200
3	0.30	QR (0.10)	0.35 ± 0.04	0.36 ± 0.04	0.37 ± 0.03	0.42 ± 0.03	0.49 ± 0.03	0.54 ± 0.04	0.58 ± 0.03	0.65 ± 0.03	0.67 ± 0.01
		QR (0.50)	0.12 ± 0.02	0.13 ± 0.02	0.17 ± 0.02	0.20 ± 0.02	0.27 ± 0.02	0.34 ± 0.02	0.41 ± 0.02	0.49 ± 0.02	0.55 ± 0.02
		QR (0.90)	0.33 ± 0.02	0.40 ± 0.02	0.42 ± 0.02	0.50 ± 0.02	0.53 ± 0.02	0.53 ± 0.02	0.61 ± 0.02	0.69 ± 0.02	0.66 ± 0.03
		GLM	0.0006 ± 0.0003	0.0012 ± 0.0004	0.0003 ± 0.0003	0.0001 ± 0.0001	0.0001 ± 0.0001	0.0004 ± 0.0002	0.0002 ± 0.0002	0.0001 ± 0.0001	0.0001 ± 0.0001
	0.50	QR (0.10)	0.13 ± 0.04	0.18 ± 0.05	0.20 ± 0.04	0.24 ± 0.05	0.26 ± 0.04	0.33 ± 0.05	0.46 ± 0.04	0.55 ± 0.03	0.57 ± 0.02
		QR (0.50)	0.03 ± 0.01	0.04 ± 0.02	0.04 ± 0.02	0.06 ± 0.02	0.10 ± 0.02	0.14 ± 0.04	0.21 ± 0.04	0.30 ± 0.03	0.42 ± 0.03
		QR (0.90)	0.12 ± 0.03	0.18 ± 0.03	0.22 ± 0.03	0.26 ± 0.02	0.31 ± 0.03	0.36 ± 0.02	0.44 ± 0.03	0.57 ± 0.03	0.57 ± 0.03
		GLM	0.0082 ± 0.0021	0.0063 ± 0.0024	0.0022 ± 0.0005	0.0019 ± 0.0006	0.0016 ± 0.0007	0.0008 ± 0.0003	0.0004 ± 0.0002	0.0001 ± 0.0001	0.0001 ± 0.0001
100	0.30	QR (0.10)	0.18 ± 0.02	0.22 ± 0.02	0.24 ± 0.03	0.30 ± 0.02	0.33 ± 0.03	0.43 ± 0.03	0.47 ± 0.03	0.56 ± 0.03	0.67 ± 0.03
		QR (0.50)	0.07 ± 0.02	0.08 ± 0.02	0.10 ± 0.02	0.16 ± 0.03	0.21 ± 0.04	0.24 ± 0.02	0.36 ± 0.02	0.44 ± 0.03	0.56 ± 0.02
		QR (0.90)	0.23 ± 0.03	0.25 ± 0.02	0.26 ± 0.02	0.33 ± 0.03	0.41 ± 0.04	0.46 ± 0.03	0.54 ± 0.03	0.63 ± 0.03	0.73 ± 0.03
		GLM	0.0192 ± 0.0068	0.0003 ± 0.0003	0.0001 ± 0.0001	0.0000 ± 0.0000	0.0000 ± 0.0000	0.0000 ± 0.0000	0.0000 ± 0.0000	0.0000 ± 0.0000	0.0000 ± 0.0000
	0.50	QR (0.10)	0.06 ± 0.02	0.09 ± 0.02	0.11 ± 0.03	0.15 ± 0.02	0.22 ± 0.03	0.27 ± 0.03	0.36 ± 0.03	0.49 ± 0.04	0.64 ± 0.03
		QR (0.50)	0.01 ± 0.00	0.01 ± 0.00	0.02 ± 0.01	0.04 ± 0.01	0.06 ± 0.01	0.08 ± 0.01	0.15 ± 0.02	0.24 ± 0.03	0.38 ± 0.03
		QR (0.90)	0.06 ± 0.01	0.06 ± 0.01	0.08 ± 0.02	0.12 ± 0.03	0.14 ± 0.02	0.21 ± 0.03	0.34 ± 0.04	0.46 ± 0.03	0.65 ± 0.03
		GLM	0.0680 ± 0.0147	0.0055 ± 0.0028	0.0016 ± 0.0007	0.0011 ± 0.0005	0.0004 ± 0.0002	0.0000 ± 0.0000	0.0007 ± 0.0007	0.0009 ± 0.0006	0.0001 ± 0.0001

*Nº. QTL:* number of loci controlling the trait, *h*^2^ :heritability, *QR:* quantile regression, *GLM:* general linear model.

Finally, it was observed that the decrease in the heritability of the trait implies a lower power of detection of QTLs when using the GLM in all scenarios evaluated (Table 1). This result is similar to that found by^62^, in which the authors compared the detection power of true significant associations using five GWAS methods. This was done using simulated data from a Chinese soybean germplasm population with different levels of heritability (*h*^2^ = 0.20, 0.50 and 0.90) and two genetic architectures with 10 and 100 QTls. As a result, the authors observed that the detection power was dramatically reduced for all methods and scenarios evaluated when the heritability of the trait was reduced. On the other hand, this behavior was not observed when using the QR methodology. The QR obtained greater or equal powers of detection of true significant associations in scenarios with lower heritability (*h*^2^ = 0.30) regardless of the number of QTLs and sample size (Table 1). This result is interesting since it indicates that QR is an interesting methodology for GWAS studies in both low and moderate heritability scenarios.

Overall, these results indicate that using quantile regression to perform GWAS in the identification of QTLs is an interesting approach. QR proved to be efficient both in scenarios with many individuals and in scenarios with a reduced population size. Additionally, this methodology also proved to be interesting for GWAS studies in which the traits have low and moderate heritabilities.

## Conclusion

The use of Quantile Regression models in genomic association studies on simulated data proved to be effective. Since its use, it allows a high power of detection of QTLs in all the scenarios analyzed in relation to the GLM. In scenarios with larger population sizes, the QR in the extreme quantiles (τ = 0.1 and 0.9) were the most efficient models in the simulated conditions because they were the ones that obtained the highest QTL detection powers. In the scenario where the detection power of the QR in the three evaluated quantiles was equal, the QR (0.50) was more efficient, as the false positive rate was lower. In the low heritability scenarios, QR obtained a high detection power of QTLs. The false positive rate obtained by the QR methodology in the scenarios with many individuals is relatively low. QR proved to be efficient both in scenarios with many individuals and in scenarios with a small population size.

## Data Availability

The datasets generated during and/or analysed during the current study are available from the corresponding author on reasonable request.
